# Supplementary material for: Declining genetic polymorphism of the C-terminus Merozoite Surface Protein-1 amidst increased Plasmodium knowlesi transmission in Thailand
Source: Malar J. 2024 Nov 13;23:342. doi: 10.1186/s12936-024-05162-z (PMC11562464; doi:10.1186/s12936-024-05162-z)
Supplement: Supplementary file 1 — Supplementary Material 1. [file 12936_2024_5162_MOESM1_ESM.docx]

**S1** List of *P. knowlesi* recent samples collected between 2018 – 2023

| **ID** | **Year** | **Collection site** | **Region** | **Reference** |
| --- | --- | --- | --- | --- |
| PK25 | 2018 | Songklah | Southern Thailand | This study |
| PK26 | 2018 | Songklah | Southern Thailand |  |
| PK27 | 2018 | Narathiwat | Southern Thailand |  |
| PK28 | 2018 | Uthai Thani | Central Thailand |  |
| PK29 | 2018 | Uthai Thani | Central Thailand |  |
| PK30 | 2018 | Uthai Thani | Central Thailand |  |
| PK31 | 2018 | Uthai Thani | Central Thailand |  |
| PK32 | 2018 | Ratchaburi | Central Thailand |  |
| PK33 | 2019 | Surat Thaini | Southern Thailand |  |
| PK34 | 2020 | Songklah | Southern Thailand |  |
| PK35 | 2021 | Narathiwat | Southern Thailand |  |
| PK36 | 2021 | Yala | Southern Thailand |  |
| PK37 | 2022 | Songklah | Southern Thailand |  |
| PK38 | 2022 | Songklah | Southern Thailand |  |
| PK39 | 2022 | Songklah | Southern Thailand |  |
| PK40 | 2023 | Yala | Southern Thailand |  |
| PK41 | 2023 | Yala | Southern Thailand |  |
| PK42 | 2023 | Yala | Southern Thailand |  |
| PK43 | 2023 | Yala | Southern Thailand |  |
| PK44 | 2023 | Yala | Southern Thailand |  |
| PK45 | 2023 | Yala | Southern Thailand |  |
| PK46 | 2023 | Yala | Southern Thailand |  |
| PK47 | 2023 | Yala | Southern Thailand |  |
| PK48 | 2023 | Yala | Southern Thailand |  |
| PK49 | 2023 | Yala | Southern Thailand |  |

**S2** List of *P. knowlesi* former samples collected between 2000 – 2009.

| **ID** | **Year** | **Collection site** | **Region** | **Reference** |
| --- | --- | --- | --- | --- |
| A1 | 2000 | Prachuab Khirikhan | Central Thailand | Putaporntip et al., 2013 |
| CT157 | 2009 | Chantaburi | Central Thailand |  |
| CT190 | 2009 | Chantaburi | Central Thailand |  |
| CT273 | 2009 | Chantaburi | Central Thailand |  |
| BMC151 | 2007 | Prachuab Khirikhan | Central Thailand |  |
| MC128 | 2006 | Prachuab Khirikhan | Central Thailand |  |
| NR234 | 2009 | Narathiwat | Southern Thailand |  |
| NR280 | 2009 | Narathiwat | Southern Thailand |  |
| NR522 | 2009 | Narathiwat | Southern Thailand |  |
| NR600 | 2009 | Narathiwat | Southern Thailand |  |
| YL975 | 2008 | Yala | Southern Thailand |  |
| YL978 | 2008 | Yala | Southern Thailand |  |
| MNR281 | 2008 | Narathiwat | Southern Thailand |  |
| HB3 | 2008 | Narathiwat | Southern Thailand |  |
| HB63 | 2008 | Narathiwat | Southern Thailand |  |
| HB92 | 2008 | Narathiwat | Southern Thailand |  |
| HB126 | 2008 | Narathiwat | Southern Thailand |  |
| HB132 | 2008 | Narathiwat | Southern Thailand |  |
| HB144 | 2008 | Narathiwat | Southern Thailand |  |
| HB149 | 2008 | Narathiwat | Southern Thailand |  |
| HB321 | 2008 | Narathiwat | Southern Thailand |  |
| HB323 | 2008 | Narathiwat | Southern Thailand |  |
| HBP11 | 2009 | Narathiwat | Southern Thailand |  |
| HBP13 | 2009 | Narathiwat | Southern Thailand |  |

Input Data File: \\M...\pkmsp1_42.meg

Population used: Former samples

Number of sequences used: 25

Selected region: 1-456 Number of sites: 456

Total number of sites (excluding sites with gaps / missing data): 456

Sites with alignment gaps or missing data: 0

Invariable (monomorphic) sites: 426

Variable (polymorphic) sites: 30 (Total number of mutations: 30)

Singleton variable sites: 9

Parsimony informative sites: 21

Singleton variable sites (two variants): 9

Site positions: 34 135 190 218 303 333 338 358 438

Parsimony informative sites (two variants): 21

Site positions: 24 27 31 42 57 89 129 138 141 172 234 249 271

325 329 346 348 349 396 423 444

Variable sites (three variants): 0

Variable sites (four variants): 0

============ Protein Coding Region ============

Genetic Code: Nuclear Universal

Protein Coding, and Non-Coding Regions:

Number of protein coding regions (exons): 1

Number of noncoding regions (intronic and flanking regions): 0

Protein coding region, from site: 1 to 456

Total number of sites in coding regions: 456

Total number of sites excluding complex codons or codons with gaps: 456

======= Synonymous/Replacement Changes =======

Segregating sites: 30 Total number of mutations: 30

Total number of Synonymous changes: 14

24 27 31 42 57 129 135 138 141 172 234 348 396

444

Total number of Replacement changes: 16

34 89 190 218 249 271 303 325 329 333 338 346 349

358 423 438

**S3** Raw data of genetic polymorphisms on *pkmsp1_42_* sequences of the 24 *P. knowlesi* isolates in Thailand during 2000 – 2009 obtained from DnaSP v5.1 software.

Input Data File: \\M...\pkmsp1_42.meg

Population used: Recent samples

Number of sequences used: 26

Selected region: 1-456 Number of sites: 456

Total number of sites (excluding sites with gaps / missing data): 456

Sites with alignment gaps or missing data: 0

Invariable (monomorphic) sites: 435

Variable (polymorphic) sites: 21 (Total number of mutations: 21)

Singleton variable sites: 5

Parsimony informative sites: 16

Singleton variable sites (two variants): 5

Site positions: 24 89 135 234 313

Parsimony informative sites (two variants): 16

Site positions: 27 31 42 129 138 141 172 249 271 325 329 346 348

349 423 444

Variable sites (three variants): 0

Variable sites (four variants): 0

============ Protein Coding Region ============

Genetic Code: Nuclear Universal

Protein Coding, and Non-Coding Regions:

Number of protein coding regions (exons): 1

Number of noncoding regions (intronic and flanking regions): 0

Protein coding region, from site: 1 to 456

Total number of sites in coding regions: 456

Total number of sites excluding complex codons or codons with gaps: 456

======= Synonymous/Replacement Changes =======

Segregating sites: 21 Total number of mutations: 21

Total number of Synonymous changes: 12

24 27 31 42 129 135 138 141 172 234 348 444

Total number of Replacement changes: 9

89 249 271 313 325 329 346 349 423

**S4** Raw data of genetic polymorphisms on *pkmsp1_42_* sequences of the 24 *P. knowlesi* isolates in Thailand during 2018 – 2023 obtained from DnaSP v5.1 software

**S5** Malaria case numbers in Thailand from 2013 to 2023, according to the report from the Ministry of Public Health.

| **Year** | ***P. falciparum*** | ***P. vivax*** | ***P. knowlesi*** |
| --- | --- | --- | --- |
| 2023 | 492 | 15663 | 259 |
| 2022 | 254 | 9603 | 176 |
| 2021 | 61 | 3083 | 72 |
| 2020 | 192 | 3638 | 22 |
| 2019 | 638 | 4589 | 19 |
| 2018 | 816 | 5554 | 31 |
| 2017 | 1379 | 9620 | 6 |
| 2016 | 3138 | 14467 | 1 |
| 2015 | 6106 | 14252 | 0 |
| 2014 | 12645 | 19319 | 0 |
| 2013 | 15744 | 18468 | 0 |

**S6** Frequencies of mutations in *pkmsp1_42_* among former and recent populations

| **Mutations** | **Former samples (n = 24)** | | **Recent samples (n = 25)** | |
| --- | --- | --- | --- | --- |
|  | **Frequencies** | **Percentage** | **Frequencies** | **Percentage** |
| D1495N | 1 | 4.2 | 0 | 0 |
| N1513S | 4 | 16.7 | 1 | 4 |
| I1547L | 1 | 4.2 | 0 | 0 |
| I1556T | 1 | 4.2 | 0 | 0 |
| N1566K | 6 | 25 | 6 | 24 |
| Q1574K | 10 | 41.7 | 2 | 8 |
| K1584N | 1 | 4.2 | 0 | 0 |
| A1588S | 0 | 0 | 1 | 4 |
| G1592S | 5 | 20.8 | 8 | 32 |
| E1593G | 4 | 16.7 | 8 | 32 |
| Q1594H | 1 | 4.2 | 0 | 0 |
| Q1596P | 1 | 4.2 | 0 | 0 |
| A1599T | 2 | 8.3 | 8 | 32 |
| Q1600E | 6 | 25 | 8 | 32 |
| E1603Q | 1 | 4.2 | 0 | 0 |
| M1624I | 2 | 8.3 | 8 | 32 |
| K1629N | 1 | 4.2 | 0 | 0 |

**S7** Genetic diversity of *pkmsp1_42_* among former samples analyzed separately between those isolated from humans and monkeys

| **Gene** | **Site** | **N** | **S** | **η** | **π ± SD** | **k** | **H** | **Hd ± SD** |
| --- | --- | --- | --- | --- | --- | --- | --- | --- |
|  | Human | 12 | 21 | 21 | 0.018 ± 0.002 | 8.045 | 10 | 0.970 ± 0.044 |
| *pkmsp1_42_* | Monkey | 12 | 23 | 23 | 0.014 ± 0.003 | 6.227 | 7 | 0.879 ± 0.075 |
|  | **All samples** | **24** | **29** | **29** | **0.018 ± 0.002** | **8.036** | **15** | **0.942 ± 0.031** |

N, number of sequences; S, number of segregating sites; η, number of mutations; π, nucleotide diversity; k, average number of nucleotide differences; H, number of haplotypes; Hd, haplotype diversity; SD, standard deviation

**S8** Neutrality tests and recombination event of *pkmsp1_42_* among former samples analyzed separately between those isolated from humans and monkeys

| **Gene** | **Site** | **d_S_ - d_N_** | **D** | **D*** | **F*** | **Rm** |
| --- | --- | --- | --- | --- | --- | --- |
| *pkmsp1_42_* | Human | 2.70* | 0.692 | 0.531 | 0.652 | 5 |
|  | Monkey | 2.33* | -0.808 | -2.334 | -1.831 | 3 |
|  | **All samples** | **2.77*** | **0.131** | **0.022** | **0.065** | **6** |

d_S_ - d_N_, Z-test for the difference between rates of synonymous substitution (d_S_) and non-synonymous substitution (d_N_); D, Tajima’s D test; D*, Fu and Li’s D* value; F*, Fu and Li’s F* value; Rm, minimum number of recombination events

**S9** Fixation index for *P. knowlesi* in Thai and Malaysian populations based on *pkmsp1_42_*

| **POPULATION 1** | **POPULATION 2** | **F_st_** |
| --- | --- | --- |
| Central Thailand | Southern Thailand | 0.3553 |
| Central Thailand | Peninsular Malaysia | 0.3231 |
| Southern Thailand | Peninsular Malaysia | 0.1804 |

F_st_, fixation index

[ 10 20 30 40 50 60 70 80 90 100 110 120 130 140 150 ]

[ * * * * * * * * * * * * * * * ]

'H_strain' AAFNTNITDMLDSRLKKRNYFLDVLDSELNPFKYSSSGEYIIKDPYKLLDLEQKKKLLGSYQYIGASVDKDLITAKDGMEYYNKMGELYKQHLEAVNAQIKEIEASVPGEQSQLNAQKEELKKYLPFLNSIQKEYESLVNMAHTYKENLKKF

PK01 ..................................................................................K.....................................................................

PK02 ............................................................................................................SG.....TE.......................I...........

PK03 ............................................................................................................SG.....TE.......................I...........

PK04 ..................................................................................K.......K.................SG......E...................................

PK05 ..........................................................................................K.................S...........................................

PK06 ..........................................................................................K.........................E...................................

PK07 ..........................................................................................K.............................................................

PK08 .............................S..........................................................................................................................

PK09 ............................................................................................................SG......E...................................

PK10 ........................................................................................................................................................

PK11 ..................................................................................K.......K.............................................................

PK12 ..................................................................................K.......K.............................................................

PK13 ...............................................................L..................K.................N.........H.P......Q.........................N......

PK14 ..................................................................................K.......K.........................E...................................

PK15 ...........N.................S..........................................................................................................................

PK16 ........................................................................T.................K.............................................................

PK17 ..........................................................................................K.............................................................

PK18 ........................................................................................................................................................

PK19 ........................................................................................................................................................

PK20 .............................S..........................................................................................................................

PK21 ........................................................................................................................................................

PK22 ........................................................................................................................................................

PK23 ..........................................................................................K.............................................................

PK24 .............................S..........................................................................................................................

PK25 ........................................................................................................................................................

PK26 ........................................................................................................................................................

PK27 ..................................................................................K.....................S...............................................

PK28 ........................................................................................................................................................

PK29 ............................................................................................................SG.....TE.......................I...........

PK30 ............................................................................................................SG.....TE.......................I...........

PK31 ............................................................................................................SG.....TE.......................I...........

PK32 ............................................................................................................SG.....TE.......................I...........

PK33 ............................................................................................................SG.....TE.......................I...........

PK34 .............................S..........................................................................................................................

PK35 ..................................................................................K.....................................................................

PK36 ..................................................................................K.....................................................................

PK37 ..................................................................................K.....................................................................

PK38 ..........................................................................................K.............................................................

PK39 ..........................................................................................K.............................................................

PK40 ........................................................................................................................................................

PK41 ........................................................................................................................................................

PK42 ............................................................................................................SG.....TE.......................I...........

PK43 ............................................................................................................SG.....TE.......................I...........

PK44 ........................................................................................................................................................

PK45 ............................................................................................................SG.....TE.......................I...........

PK46 ........................................................................................................................................................

PK47 ........................................................................................................................................................

PK48 ..................................................................................K.....................................................................

PK49 ..................................................................................K.....................................................................

**S10** Amino acid mutations derived from nonsynonymous substitutions on *pkmsp1_42_* across a total of 49 *P. knowlesi* isolates in Thailand. (PK01 – PK24 are former samples, and PK25 – PK49 are recent samples).

**S11** Frequencies of shared haplotype in *pkmsp1_42_* between Thailand and Malaysia

| **Shared Haplotype** | **Frequenciy (%)** | |
| --- | --- | --- |
|  | **Thailand (n = 49)** | **Malaysia (n = 36)** |
| H3 | 1 (2%) | 3 (8.3%) |
| H6 | 1 (2%) | 4 (11.1%) |
| H7 | 4 (8.2%) | 1 (2.8%) |
| H12 | 1 (2%) | 3 (8.3%) |
